# Microfluidics-Based Technologies for the Assessment of Castration-Resistant Prostate Cancer

**DOI:** 10.3390/cells13070575

**Published:** 2024-03-26

**Authors:** Amel Sassi, Lidan You

**Affiliations:** 1 Institute of Biomedical Engineering, University of Toronto, Toronto, ON M5S 3G9, Canada; amel.sassi@mail.utoronto.ca; 2 Department of Mechanical and Industrial Engineering, University of Toronto, Toronto, ON M5S 3G9, Canada; 3 Department of Mechanical and Materials Engineering, Queen’s University, Kingston, ON K7L 2V9, Canada

**Keywords:** microfluidics, castration-resistant prostate cancer, circulating tumor cells, biomarkers, tumor-on-a-chip

## Abstract

Castration-resistant prostate cancer remains a significant clinical challenge, wherein patients display no response to existing hormone therapies. The standard of care often includes aggressive treatment options using chemotherapy, radiation therapy and various drugs to curb the growth of additional metastases. As such, there is a dire need for the development of innovative technologies for both its diagnosis and its management. Traditionally, scientific exploration of prostate cancer and its treatment options has been heavily reliant on animal models and two-dimensional (2D) in vitro technologies. However, both laboratory tools often fail to recapitulate the dynamic tumor microenvironment, which can lead to discrepancies in drug efficacy and side effects in a clinical setting. In light of the limitations of traditional animal models and 2D in vitro technologies, the emergence of microfluidics as a tool for prostate cancer research shows tremendous promise. Namely, microfluidics-based technologies have emerged as powerful tools for assessing prostate cancer cells, isolating circulating tumor cells, and examining their behaviour using tumor-on-a-chip models. As such, this review aims to highlight recent advancements in microfluidics-based technologies for the assessment of castration-resistant prostate cancer and its potential to advance current understanding and to improve therapeutic outcomes.

## 1. Introduction

### 1.1. Prostate Cancer

Prostate cancer (PCa) is the most prevalent non-cutaneous cancer across Europe and North America, with an estimated 1 in 8 men being diagnosed during their lifetime [1]. It is also the second leading cause of cancer-related deaths among men in the United States, accounting for over 34,500 deaths in 2022 [2] PCa is characterized by the abnormal division of cells, leading to an irregular growth of the prostate gland. Diagnosis involves the use of both the digital rectal examination and PCa screening using prostate-specific antigen (PSA) [3].

For localized PCa, the standard treatment options include prostatectomy or radiation to remove malignant cells [4,5]. However, in more advanced cases, such as metastatic disease or recurrent PCa, androgen deprivation therapy (ADT) is utilized [6,7].

### 1.2. Castration-Resistant Prostate Cancer

ADT, also known as hormone therapy, targets the overexpression of the androgen receptor (AR) on the prostate in PCa patients [8]. This overexpression, driven by AR amplification, increases the binding of ligands to the AR, resulting in a conformational change and detachment from heat shock proteins that render the receptor inactive. The receptor and ligand then enter the nucleus in a homodimerized state and bind to the androgen response element in the promoter region of various androgen regulated genes [9]. As such, the overexpression of AR leads to an increase in prostate cancer cell division.

**Figure 1:**
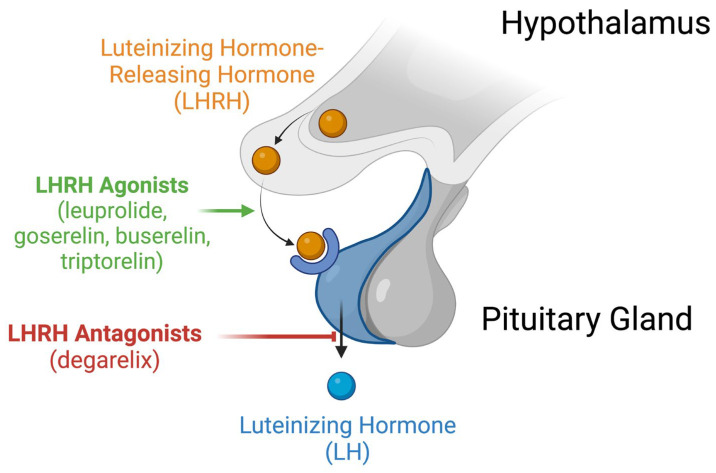
Hypothalamic–pituitary–gonadal axis and the associated effects of relevant androgen deprivation therapy medications. Sharp arrow (→) in green indicates stimulatory actions by LHRH agonists. Blunt arrow (┫) in red indicates inhibitory actions by LHRH antagonists. Created with Biorender.com.

ADT is a treatment approach that aims to decrease the level of androgens generated by the gonads. Specifically, androgen production is regulated by the hypothalamic–pituitary–gonadal axis (Figure 1).

When androgen levels are low, luteinizing hormone-releasing hormone (LHRH), commonly referred to as gonadotropin-releasing hormone (GnRH), is released from the hypothalamus and stimulates the anterior pituitary gland to produce luteinizing hormone (LH), which enters the bloodstream and the testicles to produce androgens such as dihydrotestosterone or testosterone. For the treatment of PCa, there are two major classes of ADT drugs: LHRH agonists and LHRH antagonists. Briefly, LHRH agonists (leuprolide, goserelin, buserelin and triptorelin) introduce a steady concentration of LHRH to induce an inhibitory effect on the production of LH (Figure 1) [10,11,12], whereas LHRH antagonists (degarelix) directly block the production of LH to inhibit the pathway altogether (Figure 1). Both LHRH agonists and antagonists aims to achieve “castrate” levels, which are said to occur when testosterone concentrations are less than 50 ng/mL in the bloodstream [13]. In some cases, ADT may also be combined with antiandrogens (enzalutamide, apalutamide and darolutamide) in combined androgen blockade, which blocks androgen binding to the AR and inhibit the production of androgen-regulated genes and growth [14].

While the initial response to ADT is often successful, patients with metastatic disease or advanced cases frequently develop castration resistance, characterized by disease progression or the appearance of new metastatic sites [15]. Biochemically, castration resistance is described as a 25% increase in the lowest level of PSA following treatment in patients with castrate levels of serum testosterone. Values must be confirmed by a second PSA test within 1–3 weeks, and there must be no radiological evidence of metastases [16].

### 1.3. Limitations of Current In Vitro and In Vivo Techniques

There is no existing cure for the treatment of castration-resistant prostate cancer, and therapies are often aggressive and challenging for these patients. As such, there is an urgent need to examine the behaviour of castration-resistant cancer cells in order to develop novel technologies to improve both its diagnosis and its treatment. Several existing studies are performed using in vivo animal models or traditional 2D cell culture systems. Animal models involve the use of mice inoculated with prostate cancer cells, often through intraosseous injection into the bone to induce metastasis [17,18]. However, these models lack the presence of human cells, particularly immune cells, which drastically limits the conclusions that can be made regarding human tissue responses [17]. Alternatively, conventional cell culture systems fail to recapitulate the 3D nature of organs and the various components and cells within them. The tumor microenvironment plays an essential role in the development of secondary metastatic sites. Therefore, 2D models are unable to provide information about how cancer cells interact with the extracellular matrix, other cell types or mechanical stimuli [17].

### 1.4. Microfluidic Models to Study Castration Resistance

To further elucidate the progression of prostate cancer, the field of microfluidics and thus microfluidic devices are emerging as effective tools for biomedical research. This involves a technology that processes or manipulates small amounts of fluids, using channels with dimensions ranging from tens to hundreds of micrometers [17]. In doing so, it allows the recreation of a biomimetic environment through the incorporation of various cell types and by tuning the spatiotemporal, chemical and physical gradients and the mechanical properties of the cellular microenvironment [17]. In terms of cancer metastasis, various microfluidic systems have been developed to study processes such as invasion, intravasation, extravasation and angiogenesis. Notably, Bischel et al. (2014) [19] used a microfluidic device equipped with multiphoton flavin adenine dinucleotide analysis to study prostate cancer cells (LNCaP and C4-2B) co-cultured with pre-osteoblast cells (MC3T3-E1s). The researchers began by culturing MC3T3-E1 cells to the side of the channels; following adhesion, the channels were coated with type I collagen, and prostate cancer cells were added. When C4-2B cells were co-cultured with MC3T3-E1 cells, they displayed greater migratory and invasive behaviour. Moreover, when C4-2B cells were treated with conditioned media from a mixture of C4-2B and MC3T3-E1 cells, the total percentage of protrusive C4-2B cells was increased. In summary, this study highlighted the abilities of microfluidic devices to examine the crosstalk between metastatic prostate cancer cell lines and bone cells, a key physiological process. Nevertheless, the functionality of microfluidics extends beyond co-culture devices and has been used in numerous papers to examine the physical properties of cancer cells and to isolate circulating tumor cells.

Given the complex nature of the disease, there is a need for more effective approaches for the treatment of castration-resistant prostate cancer, which will stem from a greater understanding of the various mechanisms involved. As such, this review aims to highlight recent advancements in the exploration of castration-resistant prostate cancer using microfluidics.

## 2. Microfluidic Models

### 2.1. Prostate Cancer Cell Lines Used in Current Microfluidics Systems

In vitro models play a crucial role in studying PCa, and among them, cell lines are particularly valuable. Cell lines offer a convenient and reproducible platform to investigate PCa, given their ability to metastasize to organs such as the brain, vertebrae and lymph nodes. A two-part compendium of all PCa cell lines is available, having been created by Sobel and Sadar [20,21]. Within microfluidic studies, both androgen-sensitive and androgen-insensitive PCa cell lines have been extensively examined, with prominent examples including LNCaP, DU-145 and PC3.

#### 2.1.1. Lymph Node Carcinoma of the Prostate (LNCaP)

Initially isolated from a metastatic adenocarcinoma in the lymph node of a 50-year-old Caucasian male, the LNCaP cell line is one of the most widely used androgen-sensitive PCa cells. LNCaP cells exhibit robust growth in culture, reaching densities up to 8 × 10^5^ cells/sq cm, with a doubling time of approximately 60 h. This cell line expresses crucial biomarkers for PCa, including AR, PSA and human kallikrein 2 (hK2) mRNA and protein. Notably, over 63 sublines of LNCaP have been generated, including C4, C4-2 and LNCaP-abl. Some of these sublines have become androgen-insensitive, such as LNCaP-abl, which is produced by the depletion of androgens in culture [20,21].

#### 2.1.2. DU-145

Derived from a brain adenocarcinoma in a 69-year-old Caucasian male with central nervous system metastasis in 1975, the DU-145 cell line exhibits an epithelial morphology and forms adherent layers in cell culture. With a doubling time of 34 h, DU-145 cells are not androgen-sensitive and do not express AR, PSA or hK2 mRNA or protein. However, it should be noted that while DU-145 cells have been widely used as a gold standard PCa cell line, they may not accurately represent the full spectrum of human disease. Specifically, it is argued that most human prostate cancer cells do express AR. Consequently, some investigators actively disfavour DU-145 cells, especially when aiming to investigate the effects of androgens on PCa [20,21].

#### 2.1.3. PC3

Established from the vertebrae of a 62-year-old Caucasian male, PC3 cells are similar to DU-145 cells, as they also lack expression of AR, PSA and hK2 mRNA and protein. Moreover, the cell line is androgen-insensitive and has been used to examine castration resistance. Despite similarities to DU-145, PC3 cells demonstrate greater metastatic potential, primarily targeting bone tissue. They exhibit characteristics resembling epithelial morphology and poorly differentiated adenocarcinoma features [20,21].

In summary, cell lines serve as indispensable tools for studying PCa in vitro and offer several advantages as experimental models. Cell lines offer a consistent and homogenous population of cells, enabling the examination of specific signaling pathways or genes relevant to PCa. The ability to propagate cell lines in culture allows long-term experiments and the generation of large quantities of cells. As such, the use of cell lines in microfluidic studies provides insight into pathogenesis and drug discovery in a quick and easy manner.

### 2.2. Microfluidic Studies Examining Physical Property of Castration-Resistant Prostate Cancer Cells

Exploring the characteristics and properties of castration-resistant prostate cancer cells provides valuable insights into the development of potential diagnostic tools for its detection and treatment. Traditional biomarkers expressed on PCa cells have been extensively studied; however, recent evidence suggests that analyzing the mechanical properties of cancer cells can serve as label-free markers with significant diagnostic potential [22,23,24]. While atomic force microscopy, magnetic twisting cytometry and other techniques are established techniques to quantify cellular mechanical properties, they are often labour-intensive, expensive, and offer low-throughput analyses [22,24]. Consequently, microfluidic chips have emerged as a cost-effective and promising platform for assessing cellular mechanical characteristics using fluid flow or geometric constrictions to deform cells (Table 1).

**Figure 2:**
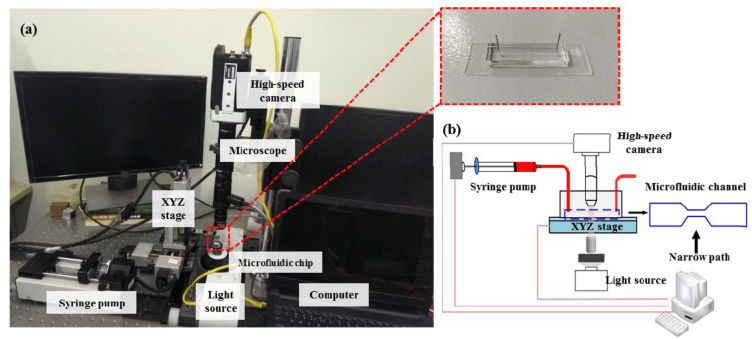
(**a**) Experimental system set-up and (**b**) schematic diagram including microfluidic device design by Liu et al. 2019 [25]. Reproduced under the terms of the CC-BY license, 2019, Liu et al. (2019), published by MDPI.

One notable example of the abovementioned microfluidic chips includes the real-time deformability cytometry developed by Otto et al. 2015 [27], whereby cells are deformed as they passed through microfluidic channel constrictions. Despite the chip’s ability to characterize a large population of cells, it was unclear whether epithelial tumor cells could flow through the bottleneck design of the channel. Notably, cancer cells possess lower elastic modulus values but are far stiffer than the red blood cells studied by Otto et al. To address this, Liu et al. (2019) [25], optimized the microfluidic chip designs to effectively to deform PCa cells, specifically LNCaP, DU-145 and PC3 cells. By increasing the cross-section of the channel by 5 μm to 25 μm × 25 μm, they enabled cells to be deformed by fluid flow shear stress rather than by contact with the channel walls (Figure 2).

**Figure 3:**
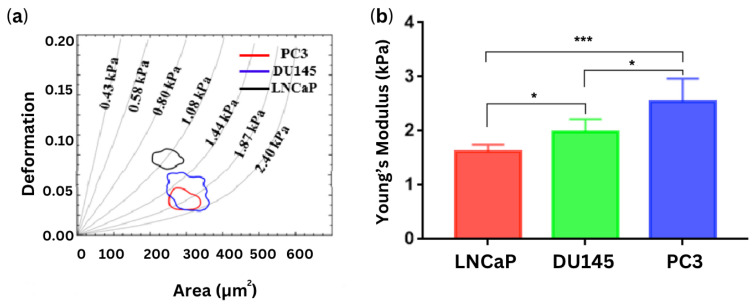
(**a**) The 50% density contour lines, representing the deformation and area of three prostate cancer cell lines with an injection rate of 0.2 μL/s (**b**) Young’s modulus of prostate cancer cell lines measured by atomic force microscopy by Liu et al. 2019. * *p* < 0.05, *** *p* < 0.001. [25]. Reproduced under the terms of the CC-BY license, 2019, Liu et al. (2019), published by MDPI.

Deformability of the cells was assessed using a high-speed camera, with optimization parameters including the microchannel size, flow rate, flow viscosity and frame rate These optimizations were critical for accurately characterizing the mechanical properties of PCa cells. The results demonstrated that the elastic modulus of the androgen-insensitive PCa cell lines (PC3 and DU-145) was higher than that of the androgen-sensitive prostate cancer cell line (LNCaP), consistent with findings obtained using atomic force microscopy (Figure 3). Namely, the apparent modulus of LNCaP cells was 1.613 ± 0.06403 kPa, while those of DU-145 and PC3 cells were 1.984 ± 0.112 kPa and 2.538 ± 0.2072 kPa, respectively [25].

However, previous studies have shown that cancer cells with greater metastatic potential exhibit less stiffness than cells with lower metastatic potential. The increased stiffness of androgen-insensitive PCa cells may be attributed to the effects of androgen deprivation therapy, which can alter cytoskeletal reorganization and promote epithelial-to-mesenchymal transition, both of which influence the mechanical properties of cells [28,29]. Namely, Wei et al. (2015) [29] discovered that greater stiffness results in increased translocation of TWIST1 through the dissociation of G3BP2, a cytoplasmic protein that renders TWIST1 inactive. The enhanced nuclear localization results in greater stiffness, which promotes epithelial-to-mesenchymal transition, tumor invasion and eventually metastasis. TWIST1 has been suggested to confer chemoresistance and is associated with poorer survival rates.

**Figure 4:**
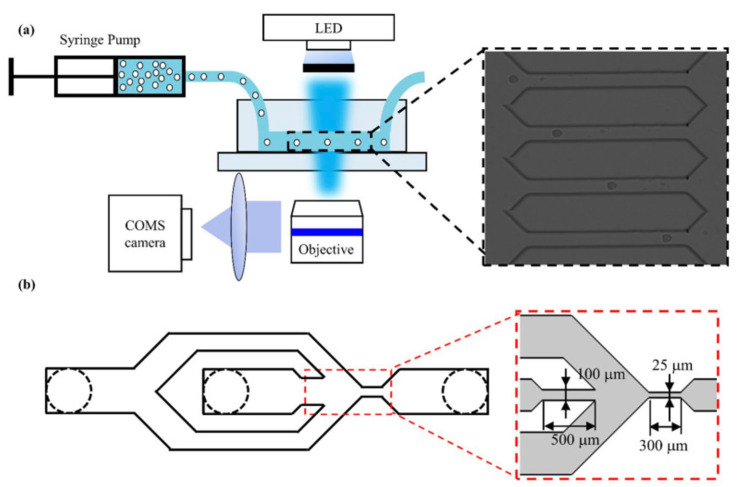
Overview of the (**a**) experimental set-up and (**b**) improved microfluidic channel geometry by Luo et al. 2021 [26]. Reproduced under the terms of the CC-BY license, 2021, Luo et al. (2021), published by MDPI.

Furthermore, Luo et al. (2021) [26] examined the physical properties of androgen-sensitive and androgen-insensitive cells treated with different concentrations of docetaxel (a microtubule-stabilizing chemotherapeutic) and enzalutamide (an antiandrogen drug). Using both LNCaP and PC3 cells, treated cells were passed through a microchannel surrounded by sheath inlets and an embedded microcolumn array at a rate of 0.2 μL/s (Figure 4).

**Figure 5:**
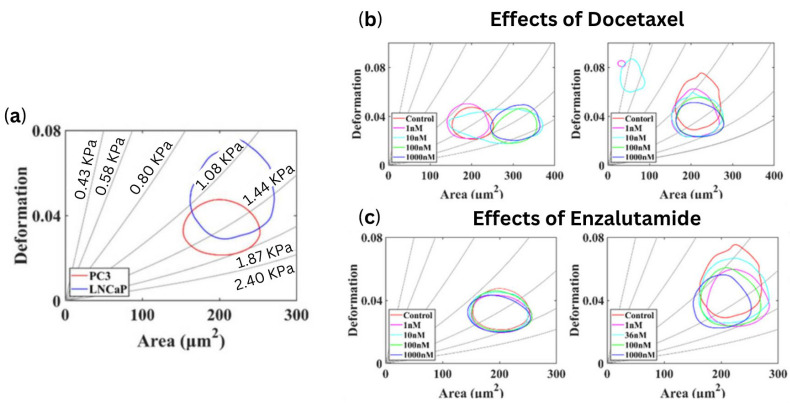
(**a**) The 50% density contour lines of PC3 and LNCaP. (**b**) The 50% density contour lines of various docetaxel concentrations for both the PC3 and LNCaP cell lines. (**c**) The 50% density contour lines of various enzalutamide concentrations for both P the C3 and LNCaP cell lines by Luo et al. 2021 [26]. Reproduced under the terms of the CC-BY license, 2021, Luo et al. (2021), published by MDPI.

As in the study by Liu et al. (2019) [25], a method to quantify deformation was developed. It was determined that PC3 cells are more stiff but smaller than LNCaP cells (Figure 5a). Upon treatment with docetaxel, PC3 cells increased in stiffness and size with increasing concentration, whereas LNCaP cells only demonstrated an increase in stiffness (Figure 5b). Alternatively, PC3 cells did not undergo any physical changes following treatment with enzalutamide, while LNCaP cells experienced changes to both size and deformation (Figure 5c).

Similarly, the authors concluded that high-throughput deformability cytometry allows the identification of androgen-sensitive and androgen-insensitive prostate cancer cells using mechanical properties as biomarkers for the identification of androgen sensitivity.

Microfluidics-based deformability cytometry provides a unique opportunity to delve into the understanding of the mechanical properties of PCa cells and drug response in cancer cells. Since mechanical properties have long been characterized as a marker for cellular cytoskeletal organization and several chemotherapeutics modify cellular mechanical properties, by exploring how the mechanical properties of PCa cells are influenced in response to drugs, microfluidics-based technologies provide insightful information on their cytotoxic effects. Through the development of a method of quantitative deformation analysis methods, microfluidic technologies offer a platform allowing a deeper understanding of the mechanical behavior of cells and its modulation by pharmacological interventions, which may improve drug design and development.

### 2.3. Microfluidic Studies Examining Circulating Castration-Resistant Prostate Cancer Cells

A significant obstacle when conducting PCa research is the failure of cell lines to accurately depict disease progression. While there are androgen-sensitive and androgen-insensitive cell lines, the transition from androgen sensitivity to the castration-resistant state and the propensity to metastasize are key features of metastatic prostate cancer [30]. Thus, to improve the clinical relevance of PCa research, circulating tumor cells (CTCs) provide valuable information and can undergo additional genetic analyses to elucidate relevant pathways. CTCs are tumor cells derived from the primary tumor or metastatic sites that circulate in the blood of cancer patients [31]. CTCs carry valuable information about the associated tumor and can be extracted non-invasively through a process known as a “liquid biopsy” [32,33]. However, isolating CTCs poses challenges due to their low concentrations in the blood, morphological similarities to white blood cells and biomarker heterogeneity.

As it stands today, Cellsearch^TM^ is the only US Food and Drug Administration-approved device for clinical CTC detection and enumeration [34]. The device targets epithelial cell adhesion molecule (EpCAM) on CTCs using ferrofluid particles coated with antibodies [35]. Cells are counted using cell image capture and analysis following immunostaining with fluorescently labeled antibodies [35]. However, the system has its limitations. It relies solely on EpCAM, thus failing to capture CTCs that have undergone epithelial-to-mesenchymal transition (EMT). Additionally, the presence of white blood cells often contaminates the sample, resulting in low purity [36]. Therefore, given the low concentration of extracted CTCs, further genetic analyses are limited. Microfluidic devices are promising in their ability to address the challenges in CTC studies. These devices utilize microfluidics-based isolation technologies to capture CTCs based on size, deformability and specific surface marker expression. In this section, we outline the advancements that have been made with regard to CTC isolation for castration-resistant prostate cancer (Table 2).

#### 2.3.1. Separation Based on Size or Deformability

Given the heterogeneity of CTCs, there has been a greater emphasis on developing label-free technologies for CTC detection and isolation based on intrinsic biophysical properties. While the studies by Liu et al. (2019) [25] and Luo et al. (2021) [26] focused on the deformability of PCa cell lines, CTC isolation requires tumor cells to be detected from a pool of red and white blood cells. The two relevant studies addressing this issue are those conducted by Renier et al. (2017) [37] and Park et al. (2016) [38].

Renier et al. (2017) [37] aimed to optimize the Vortex Chip, a vortex microfluidic technology, for improved CTC isolation based on size [37]. The device utilizes laminar microvortices to selectively enrich cells larger than red blood cells and white blood cells. Specifically, smaller cells are unable to stably reside in the laminar fluid microvortices, while larger cells are trapped, resulting in a pool of trapped CTCs. The capture efficiency ranged from 1.88 to 93.75 CTCs/7.5 mL, with purity ranging from 1.74 to 37.59%. In their study involving 22 patients with advanced metastatic castration-resistant prostate cancer, CTCs were assessed in less than 1 h. Isolated CTCs were identified through immunostaining and were defined as cells that were nucleated (DAPI+), lacked hematopoietic lineage (CD45-) and expressed PSA or cytokeratin. Interestingly, it was discovered that isolated CTCs displayed varying PSA and cytokeratin expression, with over 45.3% of cells not expressing PSA. Overall, 51% of cells were negative for epithelial markers, and this was attributed to de-differentiation and the loss of epithelial characteristics.

While size may be an effective label-free marker, CTCs derived from some tumors such as PCa overlap significantly with leukocytes. Furthermore, due to effects of ADT, castration-resistant prostate cancer cells typically experience cytoskeletal rearrangements that render them not much larger than leukocytes [38]. As such, one method to enhance the capture efficiency is isolating cells based on deformability.

**Figure 6:**
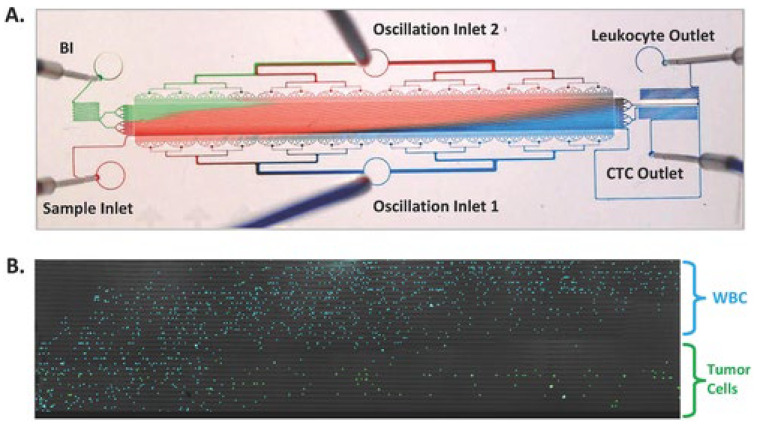
(**A**) Image of the microfluidic ratchet device by Park et al. 2016 demonstrating the flow pattern. (**B**) Fluorescent image of white blood cells (blue) and tumor cells (green) separated in the funnel matrix due to distinct sizes [38]. Reproduced with permission from Park et al. (2016); Small; published by Wiley, 2016.

In the study by Park et al. (2016) [38], a microfluidic ratchet system was developed to enhance capture efficiency based on the deformability of CTCs. This system employed continuous oscillatory flow through a matrix of tapered constrictions, creating unique flow paths for cells of similar sizes but distinct deformability (Figure 6).

**Figure 7:**
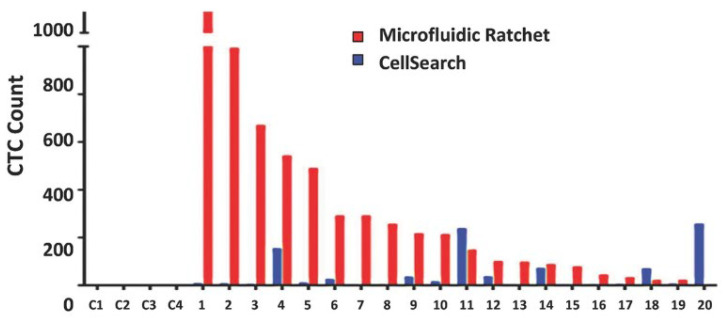
Enumeration of CTCs from 20 patients with castration-resistant prostate cancer and 4 healthy controls using the developed microfluidic ratchet device and the conventional CellSearch^TM^ System [38]. Reproduced with permission from Park et al. (2016); Small; published by Wiley, 2016.

CTCs were detected at varying levels in all 20 patients, demonstrating higher sensitivity than the CellSearch^TM^ system in detecting CTCs from castration-resistant PCa patients. The median CTC yield was 178 CTCs/7.5 mL, almost 25 times greater than that of the conventional system, which yielded only 7 CTCs/7.5 mL (Figure 7).

Both studies highlight the isolation of CTCs based on morphological and biophysical properties such as size and deformability, a method that proves to be advantageous. Following isolation, cells may be subjected to additional genetic profiling to assess disease progression or drug efficacy.

#### 2.3.2. Separation Based on Epithelial Markers

Due to the epithelial origin of cancers, the use of epithelial antigens as biomarkers to isolate CTCs was one of the first methods examined. Specifically, various devices have been developed that positively select for EpCAM, a transmembrane glycoprotein expressed in epithelial tissues, cancer stem cells, inflammatory diseases and, most importantly, epithelial cancers [46,47,48]. EpCAM is often co-localized with claudins and E-cadherins in tight junctions and at the basolateral membrane, thereby serving as an epithelial marker.

EpCAM was once thought to serve solely as a cell surface and adhesion molecule; however, in recent years, additional evidence of its role in proliferation, cancer progression and decreased survival in cancer patients has emerged [49,50,51]. Signalling commences when EpCAM is cleaved by metalloprotease tumor necrosis factor-alpha converting enzyme, which releases the intracellular domain that then interacts with components of the Wnt pathway and translocates into the nucleus, where it acts as a regulator of transcription for genes that regulate proliferation and the cell cycle [52].

In the case of microfluidic studies, the use of EpCAM as an effective biomarker was examined by Cho et al. (2021) [39], whereby a microfluidic device with immunomagnetic nanobeads bound to anti-EpCAM antibodies was developed.

**Figure 8:**
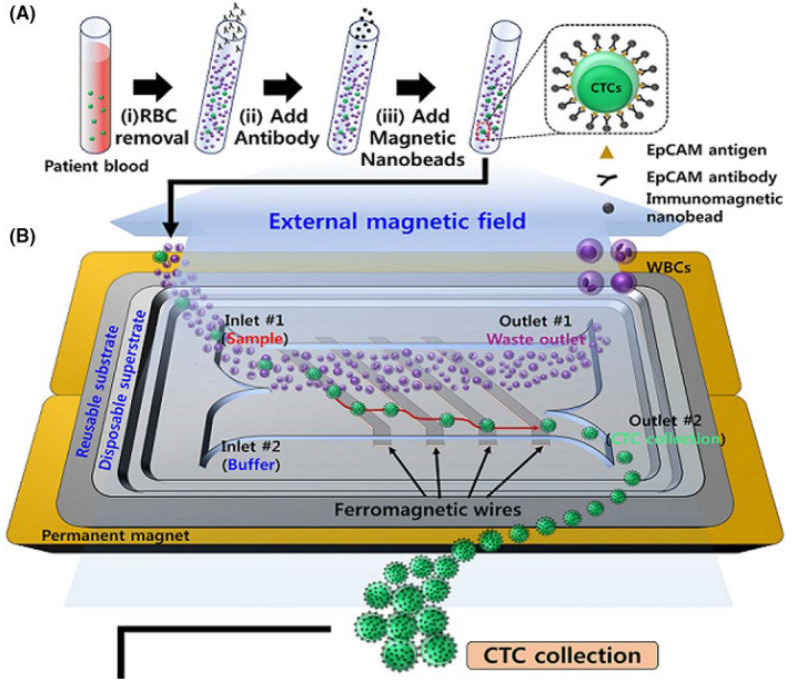
Overview of (**A**) sample preparation and (**B**) magnetophoretic microfluidic system by Cho et al. 2021 [39]. Reproduced under the terms of the CC-BY-NC-ND 4.0 license 2020; Cho et al. (2020); Cancer Science; published by Wiley, 2021.

**Figure 9:**
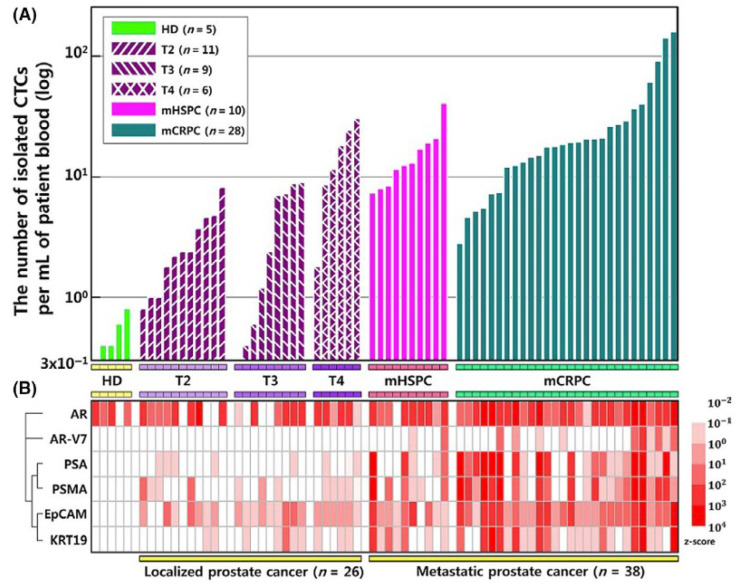
(**A**) Total number of isolated CTCs from healthy controls, metastatic hormone-sensitive prostate cancer (mHSPC) patients and metastatic castration-resistant prostate cancer (mCRPC) patients by Cho et al. 2021 [39]. (**B**) Representative heatmap, wherein columns represent individual patients and rows represent gene expression levels of isolated CTCs. Red indicates overexpression, and white indicates underexpression of the androgen receptor (AR), androgen receptor variant 7 (AR-V7), prostate-specific antigen (PSA), prostate-specific membrane antigen (PSMA), epithelial cell adhesion molecule (EpCAM) and cytokeratin 19 (KRT-19). Reproduced under the terms of the CC-BY-NC-ND 4.0 license 2020; Cho et al. (2020); Cancer Science; published by Wiley, 2021.

The device incorporates a magnetophoretic microseparator composed of inlaid ferromagnetic wires on magnets that generate a uniform external magnetic field. When the sample is passed through the microchannel, labelled CTCs are pulled towards the wires and collected in a sample tube. Investigators examined CTC isolation in patients with localized PCa, metastatic hormone-sensitive PCa and metastatic castration-resistant PCa (Figure 8). For patients with castration-resistant prostate cancer, capture efficiency averaged at 16.7 CTCs/mL of blood, while the purity was 6.7%. Cells were then subjected to genomic analysis using Droplet Digital PCR. Notably, the metastatic prostate cancer patients demonstrated a greater expression of all six genes assessed, including AR, androgen receptor variant 7 (AR-V7), PSA, PSMA, EpCAM and cytokeratin 19 (KRT-19) (Figure 9B). Additionally, patients with increased cancer progression and resistance to AR-targeting agents exhibited greater expression of AR and variants such as AR-V7.

The authors concluded that the six genes examined (EpCAM, KRT19, PSA, PSMA, AR and AR-V7) may potentially serve as genetic markers to guide early diagnosis of advanced prostate cancer. However, given the heterogeneity in EpCAM expression for patients with castration-resistant prostate cancer (Figure 9B), the use of EpCAM as a sole biomarker for CTC detection poses some concerns about the detection of PCa cells that express little to no epithelial markers following EMT.

In a study carried out by Green et al. (2019) [40], CTCs from patients with castration-resistant PCa were detected and stratified based on their expression level of EpCAM. Specifically, magnetic nanoparticles were conjugated to EpCAM antibodies, and a magnetic force was applied. Cells with greater EpCAM expression experienced greater magnetic force and were trapped in earlier zones of the microchannel, while CTCs with lower expressions were trapped in later zones. CTCs were detected in 86% of patients. Castration-resistant prostate cancer CTCs were found primarily in the low EpCAM regions of the device. Genomic analyses revealed that, in agreement with the findings by Cho et al. (2021) [39], AR-V7 expression was greatly increased in CTCs. Additionally, patients demonstrating limited response to therapy had greater levels of N-cadherin expression, a mesenchymal phenotype. The stratification method enabled the analysis of cells that are unable to be detected by CellSearch^TM^. These findings demonstrate a significant association between disease progression and mesenchymal properties and AR-V7 expression levels. Additionally, it highlights the importance of considering the heterogeneity of CTC populations in the design of microfluidic technology for the isolation of said cells.

Perhaps one of the greatest limitations of the use of EpCAM as a biomarker is that several CTCs do not have sufficient epithelial characteristics to be detected and isolated [53]. As such, the captured sample greatly skews data in favour of cells with epithelial phenotypes. This reduction in capture efficiency may largely be attributable to CTCs that undergo EMT. During this process, epithelial markers, such as EpCAM and E-cadherin, are downregulated, whereas mesenchymal markers, such as N-cadherin and vimentin, are upregulated [54]. The transformation enables cells to acquire motility and migrate from the primary tumor [55,56]. In the case of metastatic PCa, many of the CTCs will have taken on such a phenotype to carry out metastatic processes. Moreover, the dedifferentiation of cells has been associated with more aggressive properties and resistance to therapies [57]. Very little is known about the pathways implicated in this process, but findings such as those by Green et al. suggest that castration-resistant prostate cancer CTCs are associated with mesenchymal phenotypes [40].

#### 2.3.3. Prostate-Specific Markers

PSA, expressed by both normal and cancerous prostatic cells, is a serine protease that hydrolyzes proteins, such as semenogelins, produced by the seminal vesicles [58]. PSA is often considered the biomarker of choice when diagnosing PCa due to its abundance on prostate cells. According to the Canadian Cancer Society, the PSA test serves to measure the levels of PSA in the blood, wherein elevated values may indicate a PCa diagnosis [59]. However, various non-cancerous conditions may trigger a rise in PSA levels, such as prostatitis and benign prostatic hyperplasia [60,61]. As such, by 2008, the United States Preventive Series Task Force categorized the PSA test as “not recommended [62].” Despite being reclassified as “selectively offered” in 2018, various clinicians across North America approach the test with caution. When considering patients with castration-resistant PCa, PSA expression does not accurately reflect disease status [63]. In 20% of patients responsive to cytotoxic therapy, PSA expression initially rises and rapidly declines [64]. In spite of that, decline may not occur for up to 12 weeks or may not occur at all in the presence of immunomodulatory agents [65]. Therefore, depending on current and previous treatments, PSA expression may not be a reliable indicator of disease progression. Nonetheless, the longstanding use of PSA as a PCa diagnostic test has led to its exploration in serving as a biomarker for CTC isolation. Alternatively, PSMA, a transmembrane glycoprotein, has attracted interest due to its ability to stimulate proliferation in prostate cells. Existing evidence demonstrates a potential correlation between PSMA and cancer aggressiveness [66,67].

In a study carried out by Miyamoto et al. (2012) [41], the investigators aimed to explore reactivation of AR signaling in patients with castration-resistant PCa by examining PSA and PSMA expression on CTCs [41]. Namely, using a microfluidic device designed by Stott et al. (2010) [68], CTCs were isolated based on EpCAM expression and stained with antibodies for PSA and PSMA. Cells with activated AR signalling (“AR-on”) were those that were PSA-/PSMA+, “AR-off” cells were PSA+/PSMA- and “AR-mixed were PSA+/PSMA+. It was discovered that CTCs demonstrated significant intra-patient and inter-patient heterogeneity in AR signalling. The majority of CTCs from castration-resistant PCa patients were either AR-off or AR-mixed cells, with very few AR-on cells. Therefore, the authors suggested that pathways other than AR signalling may be involved in disease progression and resistance to therapies. In order to further elucidate said pathways, Miyamoto et al. (2015) [42] aimed to determine the single-cell RNA sequencing profiles of CTCs isolated from patients with prostate cancer. Following microfluidic enrichment using the CTC-iChip to magnetically deplete white blood cells and red blood cells, CTCs were identified by staining for EpCAM and mesenchymal markers. CTC heterogeneity was analyzed by comparing differences from the primary tumor and gene variations amongst patients. Over 50% of patients with castration-resistant prostate cancer had numerous AR splice variants, with 1 out of 6 cancer cells expressing various AR splice variants. Additionally, CTCs in patients who exhibited cancer progression during therapy showed enrichment for non-canonical Wnt signaling, a pathway that promotes proliferation, motility, and survival. Upon ectopic expression of the ligands for the aforementioned pathway, the survival of androgen-sensitive prostate cancer cells was enhanced following treatment with enzalutamide. Further experiments demonstrated a significant correlation between enzalutamide resistance and non-canonical Wnt signalling.

The work performed by Miyamoto et al. (2015) [42] highlights the importance of CTC capture and its potential in determining therapeutic efficacy in advanced prostate cancer. While preliminary work was concentrated on the development of CTC capture efficiency and purity, this paper extends beyond microfluidic capture and into genetic analysis of cells. As previously mentioned, it has been hypothesized that the progression of castration-resistant prostate cancer from its initial androgen-sensitive state is believed to involve additional pathways. Wnt pathways have been explored by several other investigators, including D’Abronzo et al. (2022) [69]. They present evidence that prostate cancer tumor cells differentiate into AR pathway independent lineages that are associated with an overexpression of Wntless, a Wnt transporter that promotes Wnt signalling. The authors thereby shine light on the importance of investigating the Wnt pathways as a potential target for castration-resistant prostate cancer.

#### 2.3.4. Separation Based on Multiple Characteristics

Given the complexity of castration-resistant PCa and its vast heterogeneity, microfluidic technologies reliant solely on one biomarker for the capture of CTCs often have lower capture efficiencies. As such, many studies have explored combinations of multiple characteristics to accurately identify and isolate CTCs from patient blood samples. For the detection of CTCs in patients with castration-resistant prostate cancer, many devices often incorporate selection based on size and antibodies specific to prostate cells such as PSA or PSMA.

In a study carried out by Gleghorn et al. (2010) [43], the researchers aimed to develop a microfluidic device built on principles of geometrically enhanced differential immunocapture (GEDI). GEDI incorporates flow dynamics to ensure that larger cells such as CTCs collide more frequently with the obstacles of the microchannel, all whilst including monoclonal antibodies to target PSMA to improve the isolation of CTCs. The device averaged 27 CTCs isolated from blood samples, with a capture purity of 62%. Originally published in 2010, the novelty of this study lies in the fact that enrichment is 10^9^ compared to other cells. Kirby et al. (2012) [70] continued to advance existing technology by using similar principles of GEDI with PSMA antibodies. The investigators also performed additional ex vivo drug treatment experiments by treating captured cells with docetaxel and paclitaxel and monitoring microtubule bundling. The ability to assess drug effects following isolation of CTCs highlights the potential clinical use of such microfluidic devices to assess the effects of drug regimens or additional therapies on cancer cells. These findings further emphasize the high capture efficiency and purity (68%) of the device. By the same token, Galletti et al. (2014) [44], performed ex vivo drug treatments using docetaxel or cabazitaxel, on CTCs isolated from the GEDI device developed by Kirby et al. (2012) [70]. The investigators focused their efforts on examining the effects of ERG, a transcription factor commonly overexpressed in prostate cancer [44]. It was determined that for an ETS-related gene (ERG)-negative castration-resistant prostate cancer patient, drug-target engagement was significantly enhanced in CTCs when compared to the effects on ERG-positive patients. These results suggest that ERG expression is associated with decreased taxane sensitivity. It is hypothesized that this is accomplished by ERG binding with tubulin and altering microtubule dynamics. Therefore, the efficient capture of CTCs has allowed for the identification of ERG as a potential biomarker for drug response to microtubule inhibitors.

**Figure 10:**
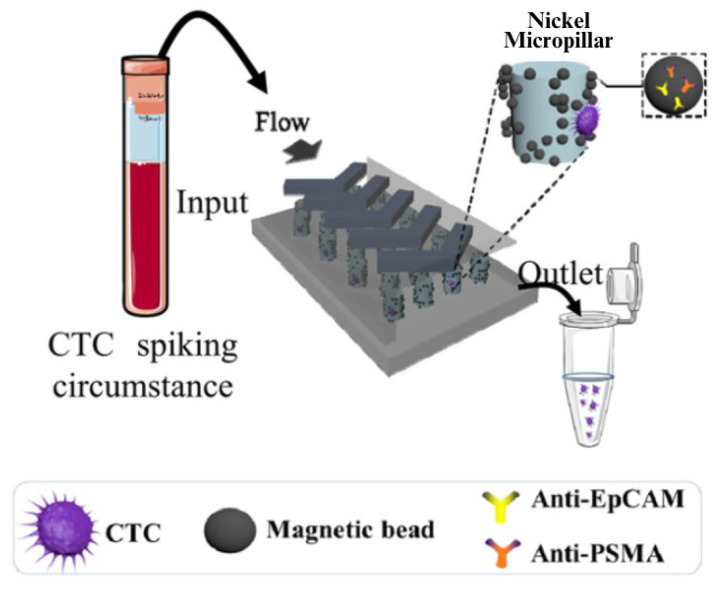
Overview of the dual-antibody-functionalized microfluidic device by Yin et al. 2018 [45]. Reprinted (adapted) with permission from Yin et al. (2018). Copyright 2018 American Chemical Society.

Alternatively, other approaches have been created to isolate CTCs based uniquely on biomarkers rather than biophysical properties such as size or deformability. In a study published by Yin et al. a dual-antibody-functionalized microfluidic device was developed to target both EpCAM and PSMA (Figure 10) [45].

**Figure 11:**
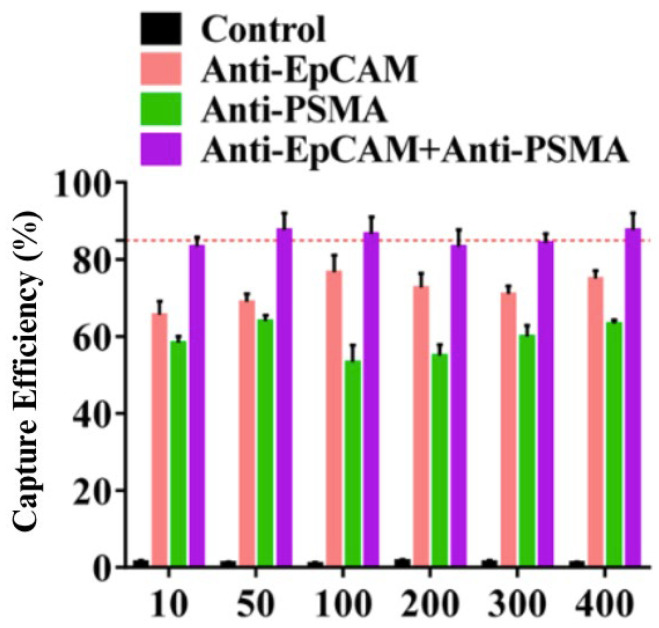
Histogram comparing capture efficiencies for increasing LNCaP cell concentrations between anti-EpCAM, anti-PSMA and dual-antibody microfluidic devices by Yin et al. 2018 [45]. Reprinted (adapted) with permission from Yin et al. (2018). Copyright 2018 American Chemical Society.

To validate the device, the authors examined the capture efficiencies on varying concentrations of LNCaP cells in blood. Using the microfluidic device, they determined the capture efficiencies when targeting EpCAM alone, PSMA alone and both EpCAM and PSMA. When comparing the total number of CTCs captured to single anti-EpCAM counterparts, the values were significantly higher, with capture efficiencies higher than 85% (Figure 11).

Moreover, when cells were analyzed using PCR, it was determined that second chromosome locus associated with prostate-1 (SChLAP1) and PSA were correlated with lymph node and bone metastases, while AR and PD-L1 may potentially be used to assess sensitivity to ADT and immunotherapy. The novelty of this device lies in its ability to detect cells that are EpCAM- and PSMA+. In sum, these dual capture systems show tremendous promise in improving capture efficiency and purity and may be effective for PCa diagnosis and the assessment of personalized therapies.

### 2.4. Tumor-on-a-Chip Models

In recent years, greater efforts have been made on developing reliable human tumor models to study cancer behaviour and the effects of existing therapies. As such, organ-on-a-chip models have emerged as novel technologies that mimic the microstructure of human organs. Tumor-on-a-chip models have gained notable interested within the field of research as a method to explore cancer biology and novel treatment options. Using microfluidics and cell culture systems, these models are designed to mimic interactions between the tumors and the affected tissues [71,72,73]. With regards to castration-resistant prostate cancer, various tumor-on-a-chip microfluidic models have been developed to assess how the cancer cells can stimulate immunity [71] and how the cells respond to chemotherapeutic drugs [72,73]. Notably, Yu et al. (2019) [71] examined the effects of both androgen-dependent and castration-resistant PCa cells on surrounding macrophages. The researchers developed a three-dimensional tissue model by stacking layers, each with its own microenvironment, together. The stacks were then used to determine how the different prostate cancer tissues alter macrophage phenotype and function. It was discovered that the castration-resistant prostate cancer drove the macrophages towards a phenotype that is more immunosuppressive, which, in turn, supports angiogenesis. To investigate the effects of chemotherapy, Pandya et al. (2017) [72] developed a 3D microfluidic platform integrated with gold electrode microsensors to monitor changes in the electrical response of DU-145 after a chemotherapeutic drug was added beside the 3D matrix. DU-145 cells showed a lower impedance change when compared to drug-susceptible and drug-tolerant cells. The study highlights a novel device that could be used to examine how cancer cells respond to drugs due to changes in electrical conductivity following cell lysis [72]. Lastly, Lin et al. (2019) [73] developed a microfabricated evolution accelerator environment to monitor how PC3 cells become resistant to docetaxel therapy using varying drug concentrations. It was determined that in areas of high drug concentration, polyploid giant cancer cells (PGCCs) would form in higher numbers that normally cannot be observed using traditional cell culture systems. The work highlights the ability of castration-resistant PCa cells to form these PGCCs, which serve as mediators of resistance, using a novel in vitro technology that is more physiologically relevant.

### 2.5. 3D-Printed Microfluidic Devices

While tumor-on-a-chip models show tremendous promise in recapitulating the physical microenvironment of cancer, soft lithography techniques require cleanrooms for fabrication and extensive manual procedures. As such, 3D bioprinting has emerged as an alternative where 3D models are fabricated using UV-curable liquid biomaterials. Steinberg et al. (2023) [74] developed a fully 3D-printed device using BV-007A resin, a luxaprint^®^ mould and FREEPRINT^®^ ortho on glass slides. They were able to demonstrate that prostate cancer spheroids, generated using PC3M-LN4 cells, were viable and maintained their growth properties. While no additional downstream analyses were carried out on PCa cells, tumor samples collected from patients with varying types of cancers, showed similar responses to chemotherapies in vitro compared to their clinical outcomes. The 3D tumor-on-a-chip model developed in this study demonstrates how the accessibility of microfluidic devices could be improved to integrate personalized medicine in the clinical setting [74].

Similarly, 3D bioprinting involves the generation of 3D models of tissue using computer graphics and biomaterials. 3D-printed microfluidic devices have emerged as cost-effective models, that allow for the creation of intricate structures with complex designs made with biological materials and cells. Unlike traditional soft-lithography methods, 3D printers are used to fabricate the microfluidic devices using inorganic or polymer materials of varying chemical compositions, stiffnesses and densities [75]. In the context of prostate cancer, Xu et al. (2023) [76], recently fabricated a co-culture system composed of a gelatin methacryloyl/chondroitin sulfate-based hydrogel to examine how hyaluronic acid and cancer-associated fibroblasts, two major components of the tumor microenvironment, influence prostate cancer behaviours. Both PC3 and C4-2b cells lines were used, which have been shown to represent castration-resistant tumors. It was discovered that hyaluronic acid promotes changes in the transcriptome of cells such that cytokine secretion, angiogenesis and epithelial-to-mesenchymal transition were upregulated. Additionally, when co-culturing PCa cells with fibroblasts, they observed an increase in cancer-associated fibroblast transformation, which was likely mediated through enhanced cytokine release. The use of a biomimetic tumor microenvironment in this study allowed for greater exploration of cell–cell interactions that may promote drug resistance and metastasis that have not been observed through traditional 2D cell culture systems.

Despite 3D printers allowing rapid fabrication of complex geometries, several 3D printers are restricted in terms of resolution when compared to soft lithography techniques, with many being limited to the macroscale [77]. Moreover, 3D-printed microchannels typically have rough surface profiles, which results in volumes of solution remaining in the device and inconsistent surfaces for cell seeding. Additionally, the composition of 3D-printing materials may reduce the concentration of proteins and lipids through absorption. Nevertheless, researchers are currently working to combine techniques by using 3D-printed devices with a layer of PDMS to enhance cell culture [78]. 3D bioprinting works to incorporate biopolymers and hydrogels to closely mimic the tumor microenvironment. However, some conventional biomaterials do not have the structural integrity for bioprinting, which necessitates additional research to enhance their microarchitecture [79]. Soft lithography provides high-resolution patterning and smoother surface properties, but limitations on its accessibility and time required for fabrication can hinder its scalability outside the laboratory environment [80].

## 3. Conclusions

In this review, we summarized the numerous functionalities of microfluidic platforms to study castration-resistant PCa. We demonstrated that microfluidic devices have been utilized to analyze intrinsic biophysical properties of PCa cell lines, as well as to address some of the limitations of existing tools for the isolation of CTCs. In the context of tumor-on-a-chip models, microfluidics allows for the creation of physiologically relevant microenvironments to study PCa behaviours and responses to stimuli. These models also offer the ability to study drug responses and interactions between tumor cells and the surrounding microenvironment, shedding light on the mechanisms underlying castration resistance and tumor progression. By analyzing the mechanical properties of castration-resistant prostate cancer cells using microfluidic chips, researchers can explore label-free markers with diagnostic potential to assess androgen sensitivity and evaluate the effects of therapeutic agents using in vitro technologies.

Moreover, the microfluidic devices highlighted in this review have proven to be effective in both capturing and isolating CTCs and have shown to confer greater advantages over traditional methods, including the FDA-approved CellSearch™ system. The devices allow the assessment of various characteristics including size, deformability and specific surface marker expression or a combination of both.

Nevertheless, certain limitations persist. With the devices presented in this review, challenges surrounding purity and the capture of CTCs with low expression levels are ongoing. To illustrate, the isolation of CTCs based on size or deformability alone may not provide the appropriate purity to carry out additional analyses. That is, the size of prostate cancer cells is not universal, and size can be altered by androgen deprivation therapy and cancer chemotherapeutics such as docetaxel. Furthermore, expression differences can occur both during and following therapy and may drastically influence the capture efficiency of CTCs. To address this, researchers are continuously exploring novel combinations of biomarkers and refining microfluidic designs to achieve greater sensitivity and accuracy in CTC isolation. To date, dual capture systems show tremendous promise in their ability to detect and isolate CTCs with varying expression patterns. Additionally, the integration of genetic analysis techniques with microfluidic CTC capture has allowed for greater understanding of the molecular basis of castration resistance and identifying potential therapeutic targets. Namely, single-cell RNA sequencing and other genomic profiling methods enable researchers to explore the heterogeneity of CTC populations.

Tumor-on-a-chip models and 3D-printed microfluidic devices offer promising avenues for studying cancer behavior and advancing personalized medicine. While tumor-on-a-chip models aim to mimic the intricate interactions between cancer cells and the surrounding microenvironment, replicating the full complexity, including the vasculature, continues to pose a challenge. As such, many of the existing models may oversimplify the dynamic nature of these interactions. Additionally, fabrication complexity may impact the translation of tumor-on-a-chip models into a clinical setting. Specifically, the processes can be complex, time-consuming and costly and require designated cleanrooms. 3D-printed microfluidic devices may bridge that gap to allow for their integration into the diagnostic or therapeutic workflow. However, limitations such as long print time and the limited choices of biocompatible materials restrict its large-scale applications. As such, further investigations are required to improve its scalability into the industry or clinical setting.

As it stands today, microfluidic technology continues to evolve rapidly, whereby advancements are driven by discoveries in materials science, nanotechnology, and biomedical engineering. Ongoing research efforts are focused on addressing limitations in CTC capture as well as fabrication accessibility by pushing the boundaries of microfluidic device capabilities. While our paper concentrates on castration-resistant prostate cancer, considerable advancements have been made in the microfluidic devices used for CTC capture for other aggressive types of cancer including triple-negative metastatic breast, pancreatic and lung cancers [81]. Equally important is the basic and bioinformatic research conducted to further stage and classify PCa. The discovery of additional biomarkers through transcriptomic and proteomic approaches introduces additional avenues to more effective CTC capture.

While research and technological advancements have the potential to revolutionize cancer care, leading to improved patient outcomes and a deeper understanding of this challenging disease, the integration of these microfluidic devices into clinical practice necessitates collaborative efforts between researchers and sustained support for these technological innovations. As improvements are made to the capture efficiencies and purity, acknowledging the policy landscape is critical to realize the full potential of microfluidic applications in castration-resistant prostate cancer research.

## Figures and Tables

**Table 1:** Microfluidic studies examining the physical properties of castration-resistant prostate cancer cells.

Reference	Cell Type	Objective	Major Findings
Liu et al. 2019 [25]	LNCaP, DU-145, PC3	Mechanically phenotype androgen-sensitive and androgen-insensitive human prostate cancer cell lines using a morphological rheological microfluidic method.	Androgen-insensitive PCa cell lines (PC3 and DU-145) exhibit high elastic modulus compared to androgen-sensitive cells (LNCaP). Intrinsic biophysical properties may serve as diagnostic markers for androgen sensitivity.
Luo et al. 2021 [26]	LNCaP, PC3	Evaluate the physical properties of androgen-sensitive and androgen-insensitive prostate cancer cell lines exposed to docetaxel and enzalutamide.	PC3 cells demonstrate increased stiffness and size with increasing docetaxel concentrations. LNCaP exhibit increased stiffness with both docetaxel and enzalutamide. Microfluidic chips can be used to evaluate the effect of therapeutic agents on androgen-insensitive PCa cells.

**Table 2:** Microfluidic studies on circulating tumor cell (CTC) isolation methods for castration-resistant prostate cancer.

Reference	Isolation Method	Biomarkers/ Properties	Capture Efficiency	Purity	Summary
Renier et al. 2017 [37]	Vortex microfluidic technology	Size	1.88–93.75 CTCs/7.5 mL	1.74–37.59%	Improved vortex chip was effective at isolating CTCs. CTCs displayed varying PSA and cytokeratin expression. 51% of cells from castration-resistant prostate cancer patient were negative for epithelial markers.
Park et al. 2016 [38]	Microfluidic ratchet system	Deformability	Median of 178 CTCs/7.5 mL	-	Detected more CTCs from castration-resistant PCa patients compared to CellSearch^TM^.
Cho et al. 2021 [39]	Immunomagnetic nanobeads bound to anti-EpCAM antibodies	Epithelial Markers	Average of 16.7 CTCs/mL	6.7%	Examined CTC isolation based on EpCAM expression. Revealed potential genetic markers for early diagnosis of castration-resistant prostate cancer.
Green et al. 2019 [40]	Magnetic particles conjugated to EpCAM antibodies	Epithelial Markers	-	-	Stratified isolated CTCs based on EpCAM expression. Correlation between AR-V7 expression and poorer response to therapy.
Miyamoto et al. [41,42]	Magnetic particles conjugated to EpCAM antibodies	Prostate-Specific Marker Staining	-	-	Identified significant heterogeneity in AR signalling in castration-resistant PCa patients. Investigated single-cell RNA sequencing profiles of CTCs and found AR splice variants and enrichment of non-canonical Wnt signalling.
Gleghorn et al. 2010 [43]	Geometrically enhanced differential immunocapture and immunocapture using PMSA antibodies	Multiple Characteristics (Size + Prostate-Specific Markers)	Average of 27 CTCs/7.5 mL	68%	Developed a microfluidic device using GEDI and PSMA antibodies and enhanced CTC capture efficiency and purity.
Galletti et al. 2014 [44]	Geometrically enhanced differential immunocapture and immunocapture using PMSA antibodies	Multiple Characteristics (Size + Prostate-Specific Markers)	-	-	Carried out ex vivo drug treatment experiments on captured CTCs. Suggested that ERG may be used as a biomarker for drug response to microtubule inhibitors.
Yin et al. 2018 [45]	Dual-antibody-functionalized microfluidic device (EpCAM and PMSA)	Multiple Characteristics (Epithelial Markers + Prostate-Specific Markers)	6.6 CTCs/2 mL	-	Demonstrated correlations between genetic markers (SChLAP1, PSA, PSMA, AR and PD-L1) and clinical features in PCa patients.

## Abbreviations

2D	Two-dimensional
PCa	Prostate cancer
PSA	Prostate-specific antigen
ADT	Androgen deprivation therapy
AR	Androgen receptor
LHRH	Luteinizing hormone-releasing hormone
GnRH	Gonadotropin-releasing hormone
LH	Luteinizing hormone
LNCaP	Lymph node carcinoma of the prostate
PCR	Polymerase chain reaction
hK2	Human kallikrein 2
CTC	Circulating tumor cell
EMT	Epithelial-to-mesenchymal transition
EpCAM	Epithelial cellular adhesion molecule
PSMA	Prostate-specific membrane antigen
AR-V7	Androgen receptor variant 7
KRT19	Keratin 19
GEDI	Geometrically enhanced differential immunocapture
ERG	ETS-related gene
SChLAP1	Second chromosome locus associated with prostate-1
